# Editorial: Intelligent Systems for Genome Functional Annotations

**DOI:** 10.3389/fgene.2020.00915

**Published:** 2020-08-25

**Authors:** Shandar Ahmad, Pedro J. Ballester, Michael Fernandez

**Affiliations:** ^1^School of Computational and Integrative Sciences, Jawaharlal Nehru University, New Delhi, India; ^2^Cancer Research Center of Marseille, INSERM U1068, Marseille, France; ^3^Institut Paoli-Calmettes, Marseille, France; ^4^Aix-Marseille Université, Marseille, France; ^5^CNRS UMR7258, Marseille, France; ^6^Department of Urologic Sciences, Faculty of Medicine, Vancouver Prostate Centre, University of British Columbia, Vancouver, BC, Canada

**Keywords:** functional annotation, protein-protein interaction (PPI), machine learning, gene annotation, intelligent system applications

Functional annotation of an entire genome is critical to the understanding of any biological process or pathway. Yet, large parts of the human genome, and much more in the non-model organisms, remain without annotations. Simple, sequence-similarity based annotations have been found to be grossly inadequate for this purpose. More complex models, often based on intelligent systems, such as Machine Learning (ML) have proved to be very helpful. Indeed, ML models have key properties that makes them particularly useful for genome annotation (Yip et al., 2013). In their basic formulation, ML techniques have found their way into the field of biological functional annotation quite early. For example, secondary structure prediction using ML was carried out as early as in mid-1980's and many other areas of biological sequence, structure and/or function prediction have seen great advances in terms of the complexity of techniques, feature engineering, and other principles of data-driven analytics.

Several computational techniques have been developed exclusively for solving functional annotation problems. However, most of the growth has been in terms of the application of emerging and established computational techniques in the biological domain. ML software has often been used as a blackbox tool, while researchers focus on the biological concept of the problem and its solution. More recently, deep learning based neural networks methods have made rapid progress and have shown particular success with problems associated with large amounts of biological data and complex system representations. Typically popular amongst them have been convolutional neural networks (CNN), multi-layer perceptrons (MLP) and long short-term memory networks (LSTM), with widely different forms and learning strategies. On the other hand the biological understanding of molecular function and organization of knowledge on this subject has also undergone rapid advances. Instead of scattered and ambiguous labeling of function, systematic annotations in terms of biological (disease, gene, and protein etc.) ontologies with hierarchical and nested labels from semantic models have made the task of annotation learning and prediction of biological function much more robust. Clearly, much has been achieved on biological and technical aspects of functional annotations, but many hurdles remain. It was under this context, this special issue was proposed to promote the reporting of various aspects of biological functional annotations where different types of intelligent systems/ML have been used to solve functional annotation problems.

In the eight research papers forming this special issue, a special aspect of functional annotation i.e., for predicting drugability was reported. There is a need to annotate gene products to indicate whether these are likely to be druggable or not. Ghadermarzi et al. argued that the majority of the druggable human proteome is yet to be annotated and explored. To advance on that front, these authors collected the data from three types of protein targets: druggable, non-druggable, and possibly druggable. Both new and established markers for each protein were extracted from its protein sequence or names/identifiers. They discovered that the possibly druggable proteins have significantly higher abundance of alternative splicing isoforms, relatively large number of domains, higher degree of centrality in the protein-protein interaction networks, and lower numbers of conserved and surface residues, when compared with the non-druggable proteins. These markers can be helpful to find novel druggable human proteins and provide interesting insights into the cellular functions and subcellular locations of both current drug targets and potentially druggable proteins.

The genome can also be annotated with those regions whose alterations in tumor cells are found to control patient response to a drug treatment. Thus, the contribution from Bomane et al. looked at this problem from a precision oncology perspective. In particular, authors investigated the extent to which it is possible to predict breast cancer patient response to the mitotic inhibitor paclitaxel using the US National Cancer Institute's Genomic Data Commons. These datasets comprised the responses of breast cancer patients to paclitaxel along with six molecular profiles of their tumors. Ten ML algorithms were applied to each of these profiles and the resulting 60 classifiers evaluated on the held-out patients. Only three of these 60 models were at all predictive, highlighting the crucial importance of a broad search to avoid suboptimal results. DNA methylation and miRNA profiles were the most informative overall. In combination with these two profiles, the ML algorithms selecting the smallest subset of molecular features were found to generate the most predictive classifiers.

In addition to supervised ML models for function annotation, unsupervised ML methods have shown to be extremely successful to interpret experiments when labeled experiments are not available. For example, annotations from newly invented single-cell RNA sequencing (scRNA-seq) technology (Sasagawa et al., 2019) is incredibly challenging because of the lack of labels for individual cells. Purely unsupervised clustering methods, such as t-distributed stochastic neighbor embedding and uniform manifold approximation and projection, have been employed to obtain low-dimensional embedding of cell–cell relationships with the foreseeable drawback of highly dependent upon genes selected for clustering. Taguchi and Turki contribution explored tensor decomposition (TD)-based unsupervised feature extraction (FE) (Taguchi, 2019) to integrate two scRNA-seq expression profiles that measure human and mouse midbrain development. TD-based unsupervised FE showed to be a promising method to effectively integrate two scRNA-seq profiles while outperforming other popular unsupervised selection methods.

The integration of biological experiments also requires informatics tools and platforms that combine and analyse different sources of biological data (Triplet and Butler, 2014). TargetMine is an integrative data analysis platform for target prioritization and broad-based biological knowledge discovery. The recent improvement of the platform described by Chen et al. forms a contribution in that direction and highlights newly modeled biological data types and the improvement of new analytical and visual tools. Enhanced coverage of gene–gene relations, and small molecule metabolite to pathway mappings are now implemented in TargetMine together with an improved literature survey feature. The platform also incorporated *in silico* predictions of gene functional associations such as protein–protein interactions and global gene co-expression. Authors demonstrated how the newer enhancements in TargetMine provides a more expansive coverage of the biological data space and can help interpret genotype–phenotype relations.

Finding new biological targets is key for designing potential new drugs. Computational biology can help identifying targets by sorting the parasite's metabolic pathways that pins out proteins essential for its survival. Bora and Jha contributed a kinetic modeling for determining targets against *Leishmania donovani*, a deadly human pathogen responsible for causing *Visceral Leishmaniasis*. Metabolic pathway and Protein-Protein Interactions (PPI) were integrated to analyse the “purine salvage” pathway, which is mandatory for parasite survival. Available experimental data was used to develop a kinetic model of Purine salvage pathway that helped marking of crucial enzymes involved in the synthesis of the metabolites. Additionally, PPI analysis of the pathway assisted in building a static interaction network for selected proteins. Dynamic Modeling and Topological analysis of the PPI network through centrality measures were combined to detected targets. ADSL (Adenylosuccinate lyase) and IMPDH (Inosine-50-monophosphate dehydrogenase) enzymes appeared to be crucial and further modeling of three dimensional structure of ADSL enzyme aided toward the search for antiparasitic drugs for the treatment of *Visceral Leishmaniasis*.

In terms of specific issues discussed in this special issue, Wang et al. have reported the use of one of the top unsupervised machine learning technique known as overlapping cluster generator (OCG) for the functional characterization of hitherto poorly annotated P311. They propose that the proteins on the interface of OCGs represent multifunctional property and based on this PPI characterization propose that P311 may be involved in inflammatory responses, cell proliferation, and coagulation. While protein-wise functional annotation is a key biological problem of interest, more detailed characterization of proteins to gain general insights into their structure and function are critically important. In this regards, amino acid repeats in proteins play an important role in their structures and by consequence their functions. Thus, Rajathei et al. have reported an analysis of protein repeat regions and their role in structure and function of proteins. They also report that repeat regions of longer than 15 residues are present in about 67% of proteins in the Uniprot, when viewed in a non-redundant manner. Biological function annotation cannot be complete without looking at the sensitivities at the epigenetic and single nucleotide polymorphism-driven functional diversity. Database and resources form the centerstage in any such analysis. Ma et al. have presented a database FeatSNP that focuses specifically on the SNPs in epigenetic factors in human brain. In the absence of a thorough understanding of human brain and proteins involved in performing cognitive and behavioral functions, such a database will be of immense value for people working on understanding genomic perspectives of protein function in brain and its related disorders.

Overall, the special issue covered various aspects of ML both supervised and unsupervised with classical clustering, overlapping clustering and general statistical principles, neural networks, tensor decomposition and related techniques. The biological systems investigated included generalized druggability, P331 systems, brain associated proteins and *Leishmania donovani*. Thus, special issue brings together typical systems in which ML and intelligent systems have helped gaining predictive value and biological insights into the vast area of biological function annotation. We hope the readers from computational biology and the domain specific researchers will be benefitted by reading articles included in this special issue.

## Author Contributions

All authors listed have made a substantial, direct and intellectual contribution to the work, and approved it for publication.

## Conflict of Interest

The authors declare that the research was conducted in the absence of any commercial or financial relationships that could be construed as a potential conflict of interest.
